# Overcoming Intrinsic and Acquired Resistance Mechanisms Associated with the Cell Wall of Gram-Negative Bacteria

**DOI:** 10.3390/antibiotics9090623

**Published:** 2020-09-19

**Authors:** Rachael E. Impey, Daniel A. Hawkins, J. Mark Sutton, Tatiana P. Soares da Costa

**Affiliations:** 1 Department of Biochemistry and Genetics, La Trobe Institute for Molecular Science, La Trobe University, Melbourne, VIC 3086, Australia; reimpey@students.latrobe.edu.au (R.E.I.); daniel.hawkins@latrobe.edu.au (D.A.H.); 2 National Infection Service, Research and Development Institute, Public Health England, Porton Down, Salisbury, Wiltshire SP4 0JG, UK; mark.sutton@phe.gov.uk

**Keywords:** antibiotic discovery, antimicrobial resistance, cell wall, Gram-negative bacteria

## Abstract

The global increase in multi-drug-resistant bacteria is severely impacting our ability to effectively treat common infections. For Gram-negative bacteria, their intrinsic and acquired resistance mechanisms are heightened by their unique cell wall structure. The cell wall, while being a target of some antibiotics, represents a barrier due to the inability of most antibacterial compounds to traverse and reach their intended target. This means that its composition and resulting mechanisms of resistance must be considered when developing new therapies. Here, we discuss potential antibiotic targets within the most well-characterised resistance mechanisms associated with the cell wall in Gram-negative bacteria, including the outer membrane structure, porins and efflux pumps. We also provide a timely update on the current progress of inhibitor development in these areas. Such compounds could represent new avenues for drug discovery as well as adjuvant therapy to help us overcome antibiotic resistance.

## 1. Introduction

Antimicrobial resistance (AMR) represents one of the biggest threats facing modern medicine. If no action is taken, it is predicted that AMR will result in 10 million fatalities by the year 2050, which will largely be attributed to infections caused by Gram-negative bacteria (GNB) [1]. Depending on the level of resistance, GNB can be classified as either multi-drug resistant (MDR), extensively drug resistant (XDR) or pan-drug resistant (PDR), the latter displaying resistance to all antibiotic classes [2]. Recent studies have estimated that some nosocomial populations contain up to 34% XDR bacteria [3], with rising levels of XDR *Pseudomonas aeruginosa* being reported that are resistant to carbapenems as the last-resort antibiotic [4]. An alarming increase in clinical resistance has also been observed in *Acinetobacter baumannii* and *Klebsiella pneumoniae* [5]. This aligns with priority pathogen reports by the Centres for Disease Control and Prevention and the World Health Organisation, which highlight the urgent need for new antibiotics against these drug-resistant GNB [6,7]. Despite this, most new antibiotics, defined here as those that are in late-phase clinical trials, are variants of existing drugs or combinations of β-lactams and β-lactamase inhibitors, and as such, may still be subject to iterations of pre-existing resistance mechanisms [8].

One of the key mechanisms limiting antibiotic efficacy in GNB is the presence of a complex cell wall, consisting of an outer membrane and cytoplasmic membrane with differing physicochemical properties, which are separated by a cross-linked peptidoglycan layer. This review will focus on the role that the cell wall plays in both intrinsic and acquired resistance mechanisms that limit the efficacy of many antibacterials in GNB, and the emerging novel strategies that are being developed to overcome them. Many of these focus on disrupting the complex mechanisms involved in the synthesis, assembly and maintenance of the dual-membrane system, which is essential to the integrity of GNB cells. Whilst this system is one of the major contributors to antibiotic resistance in GNB, there is a growing awareness that this might also be an Achilles’ heel and provide important new targets for therapy.

## 2. Outer Membrane

Unlike Gram-positive bacteria, GNB have a hydrophobic outer membrane bilayer, containing lipopolysaccharide (LPS) molecules, phospholipids and outer membrane proteins (OMPs), including pore-forming proteins [9]. As such, this dynamic outer membrane acts as a barrier to several antibiotics that are typically effective against Gram-positive bacteria, such as vancomycin [10]. Most antimicrobials must traverse this outer membrane to reach their targets. Hydrophobic antibiotics, such as chloramphenicol and aminoglycosides, utilise a diffusion pathway through the lipids, but other hydrophobic and polar molecules may interact with LPS and have restricted entry [9,11]. Alterations of the outer membrane permeability influence the diffusion of hydrophobic antibiotics, which can result in poor uptake. For instance, in *P. aeruginosa*, mutations in the LPS transport pathway decreases membrane permeability, resulting in increased tobramycin resistance [11]. Hydrophilic antibiotics, including β-lactams, use channel proteins such as porins to gain entry into the periplasm [9,12], but molecules with cytoplasmic targets are restricted from entering the cytoplasm due to the inner membrane.

Alternatively, polymyxins and some cationic peptides exert their effects on the outer layer of the membrane itself by binding to the LPS and phospholipids, which destabilises the inner cell membrane [13]. Many bacteria that acquire polymyxin resistance through exposure to therapy, including *K. pneumoniae*, *Salmonella enterica, A. baumannii* and *P. aeruginosa*, exhibit overexpression of the *pmrCAB* operon (or species equivalent) [14,15,16,17,18]. One of the gene products, phosphoethanolamine transferase (PmrC), is responsible for the modification of lipid A [14,15,16,17,18]. This alteration lowers the negative charge of the LPS, resulting in varying levels of resistance to colistin and other polymyxins [14,15,16,17,18]. The regulatory complexes PmrAB, a two-component system (TCS) directly responsible for the regulation of PmrC, and PhoPQ have also been implicated in other lipid A resistance mechanisms [13,19,20]. This includes the addition of l-4-aminoarabinose, which reduces the dependency of LPS on the cross-bridging cations for stabilisation, and thus, blocks the self-promoted uptake ability of polymyxins [13,15]. The *pmrCAB* operon and/or the *arn* operon, which also mediate lipid modification, are proposed to play a role in intrinsic resistance to polymyxins in a number of clinical pathogens, including *Proteus mirabilis*, *Serratia marcescens*, *Burkholderia* spp. and *Yersinia* spp. [21,22,23]. The regulators of these lipid modifying enzymes mediate cell responses to different stresses and influence both drug resistance and virulence. Worryingly, these *pmrCAB* mutations can cause heteroresistance to polymyxins, including colistin. Heteroresistance occurs when a subpopulation of bacteria show resistance to certain antibiotics within a system that is generally considered to be susceptible [24,25]. Such phenomenon is often associated with breakdown in treatment and can lead to the proliferation of the resistant subpopulation and the emergence of a stable resistant strain, which may result in treatment failures [24,25]. Heteroresistance to colistin has been reported for several species, including *A. baumannii* [26], *K. pneumoniae* [27], *P. aeruginosa* [28], *Enterobacter cloacae* [29] and *Salmonella* spp. [30]. The mutations mentioned above and the role that they play in heteroresistance indicates that PmrAB could represent a relevant target for inhibitor development, which will be discussed below.

Significant interest has been focussed on another mechanism of acquired polymyxin resistance, mediated by a **m**obile **c**olistin-**r**esistance gene (*mcr-1*) and variants. This was originally described in *Escherichia coli* [31], but has been widely reported in other GNB isolates collected both before and after the original report [32]. The biochemical mechanism of resistance is similar to that mediated by PmrC as MCR1 protein is a probable phosphoethanolamine transferase, hypothesised to have originated from an intrinsically resistant environmental strain (e.g., *Paenibacillus* spp.), but now disseminated globally due to its presence on a highly transmissible plasmid [31]. Another polymyxin resistance mechanism only described in *A. baumannii* is the complete loss of the LPS, mediated by disabling mutations in one of the *lpxACD* genes [33]. Further examples of outer membrane resistance include the up-regulation of the OMP OprH in *P. aeruginosa*, contributing to both aminoglycoside and colistin resistance [34], as well as resistance mediated by mutations in regulators of membrane stress responses in a variety of species [35,36,37].

Targeting outer membrane permeability has been considered an underexploited approach to enhance antibiotic susceptibility and prolong the effectiveness of first-line treatments in the clinic. Current treatments that improve membrane permeability based on synergy between licensed antibiotics involve either polymyxin B or colistin (polymyxin E) [38]. While polymyxins have shown promising synergistic effects in vitro against GNB, the overall concern lies with their associated nephrotoxic effects [39]. Despite this, MDR *A. baumannii* infections still rely heavily on the use of polymyxins as a primary treatment given the lack of alternative effective therapeutics [39]. There has been renewed interest in developing further polymyxin derivatives as candidate therapies, with considerable work taking place to optimise efficacy, reduce cytotoxicity and address issues with intrinsic and adaptive resistance [40].

**Figure 1:**
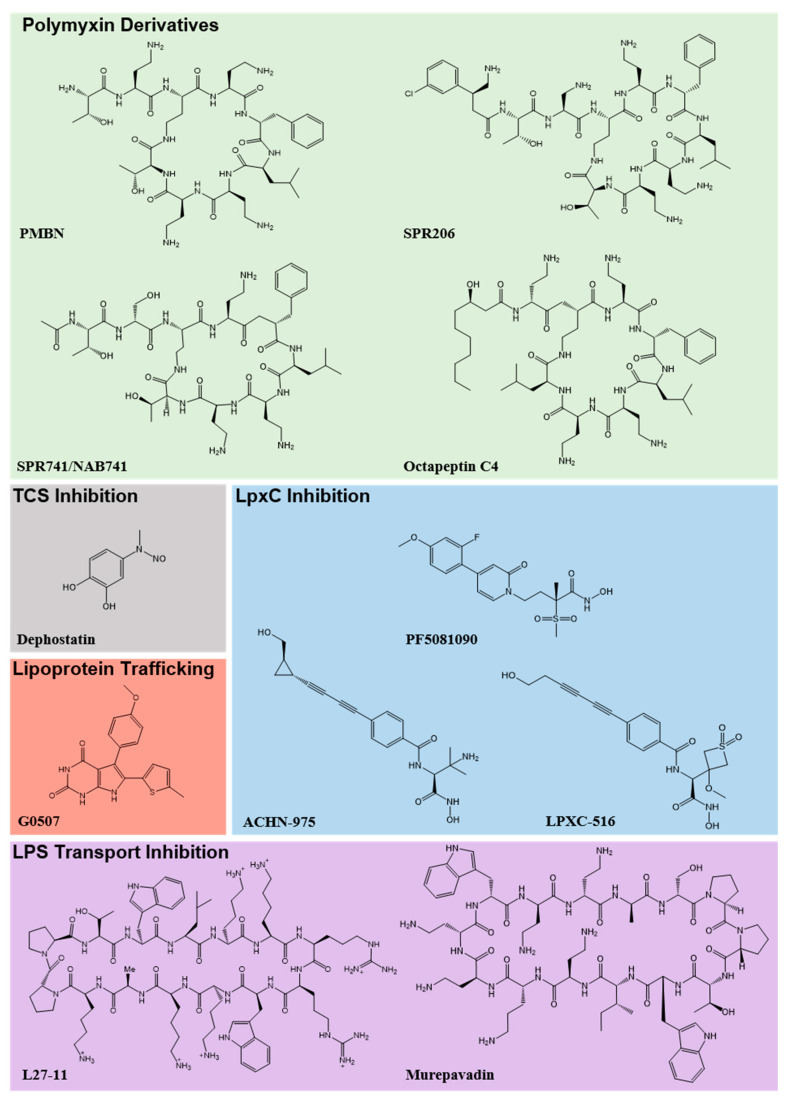
Outer Membrane Inhibitors. Polymyxin analogues (green), two-component system (TCS) (grey), lipoprotein trafficking (red), LpxC (blue) and LPS transport (purple) inhibitors. PMBN has been well characterised as a combinatorial agent that allows the disruption to the cell membrane to potentiate current antibiotics. SPR741/NAB741 and SPR206 are polymyxin B analogues that have lowered toxicity compared to the parent compound. Octapeptin C4 is structurally similar to polymyxins but has shown a slowed resistance profile compared to the parent compounds of polymyxins B and E. Dephostatin exhibits anti-virulence properties against *Salmonella* spp. by inhibiting both SsrA-SsB and PmrB-PmrA TCSs. G0507 is an inhibitor of the LolCDE ABC transporter in lipoprotein trafficking. PF5081090 and ACHN-975 were designed as LpxC inhibitors with efficacy in murine infection models against *P. aeruginosa*. LPXC-516 is an analogue of ACHN-975. L27-11 and murepavadin are macrocyclic peptidomimetic compounds that inhibit the enzyme LptD in the LPS transport pathway.

One of the most well-known membrane permeabilisers, polymyxin B nonapeptide (PMBN) (Figure 1), has been used in in vitro studies since its discovery in 1992 [41]. Despite the lack of antibacterial activity, PMBN’s ability to permeabilise the membrane allows resistant bacteria to be re-sensitised to several classes of antibiotics [41,42]. Moreover, reduced toxicity has been observed in animal models when compared to polymyxin B [43,44]. Thus, PMBN has become a valuable benchmark and building block for the development of polymyxin derivatives. Indeed, a polymyxin B analogue, SPR741 (originally named NAB741, Figure 1), displays synergy with rifampicin and clarithromycin in murine infections, and it has since completed phase I clinical trials [43,45]. Furthermore, the analogue SPR206 (Figure 1), which progressed from phase I clinical trials, displays antibacterial activity against several MDR GNB [46]. Despite historical concerns about nephrotoxicity, recent polymyxin analogues such as SPR206 do not appear to have cytotoxic effects, showing promise for future investigation into polymyxin-like compounds as potential membrane permeabilisers.

Recent development of other cyclic peptides termed octapeptins, which also permeabilise the outer membrane as at least part of their mechanism of action, has generated promising in vitro data even against MDR GNB strains that are resistant to polymyxins [47,48,49]. Octapeptins are structurally related to polymyxins, with analogues including octapeptin C4 (Figure 1) showing significant promise [49]. Indeed, where a 1000-fold reduction in antibacterial activity was observed for *K. pneumoniae* after continuous exposure to polymyxins B and E (colistin) in resistance studies, only a 4-fold decrease was seen for octapeptin C4 in the same timeframe [47]. Although a great effort has been made to elucidate the mode of action of these octapeptins, further investigation is needed to fully explore their potential as antibiotic treatments or adjuvants [47,48,49]. Recently, the non-profit funder, Carb-X, invested heavily in the ongoing research into octapeptins [50]. In other related strategies, an attempt to overcome already existing polymyxin resistance has been pursued through the investigation of the small molecule inhibitor dephostatin (Figure 1) [51]. Dephostatin has been shown to disrupt the signalling of TCSs, including PmrAB as previously described, and in doing so, re-sensitises *Salmonella* spp. to colistin and reduces virulence in both cell-based assays and animal models [51].

Alternative strategies have looked at targeting the major component of the outer membrane, the LPS layer. There are two main approaches for targeting the LPS layer—lipid A biosynthesis or LPS transport to the outer membrane. LpxC is the best characterised enzyme and a key regulator of lipid A biosynthesis, which is generally conserved across all GNB [52]. Research into LpxC inhibitors began before the discovery of the enzyme. However, early attempts were abandoned due to the lack of broad-spectrum activity [52]. Two lead compounds, PF5081090 and ACHN-975 (Figure 1), were developed by Pfizer and Achaogen, respectively, with both showing activity against *P. aeruginosa* in murine models [53,54]. ACHN-975 (Figure 1) is the only known LpxC inhibitor to have completed phase 1 clinical trials, but has not progressed further [55]. Analogues of ACHN-975 have been developed since, with LPXC-516 (Figure 1) showing the most promising activity against GNB, including *Enterobacteriaceae* [56]. However, concerns over cardiovascular toxicity have halted the development of these compounds [56].

In *A. baumannii*, mutational studies of the lipid biosynthetic enzymes LpxA, LpxC and LpxD unexpectedly revealed that LPS was not essential for growth, at least in this organism. On the contrary, a loss of functional LPS enzymes resulted in increased resistance to azithromycin, rifampicin and vancomycin [57]. Although mutations in these genes have resulted in colistin resistance [33,58], they have also been associated with a loss of fitness. This is linked to avirulence in a *Galleria melonella* model, coupled with increased biocide susceptibility [58]. Based on such observations, the LpxC inhibitor, PF5081090 (Figure 1), has been suggested as an anti-virulence strategy and repurposed as a synergistic agent to re-sensitise *A. baumannii* to current antibiotics through the permeabilisation of the outer membrane [59].

Beyond the biosynthetic pathway, the translocation of lipid A across the cytoplasmic membrane and transport of the LPS to the outer membrane mediated by the multi-protein Lpt complex are also attractive targets. Inhibition of this pathway was shown using a macrocyclic peptidomimetic compound (L27-11, Figure 1) that targets the LptD enzyme on the outer membrane [60], and displays antibacterial activity against *P. aeruginosa* [61]. Other macrocyclic peptides have also been used to target LptD in clinical trials. Murepavadin (Figure 1) demonstrated excellent in vitro efficacy against >1000 clinical MDR *P. aeruginosa* strains before successfully completing phases I and II clinical trials [62]. Investigation into intravenous administration was halted at phase III due to increased hepatic toxicity [63]. However, preclinical research into inhaled murepavadin is ongoing. Harrison et al. recently demonstrated that other mutants in this transport pathway resulted in aminoglycoside hypersensitivity in *P. aeruginosa* [64]. This study noted that mutations in the LptG enzyme could potentially interfere with the dimerisation of its LptB counterpart [64], highlighting its potential as a novel antibiotic target. A recent study has examined the role of the membrane transporter PbgA as a target for drug development and have identified its encoding gene to be essential for LPS biogenesis in *E. coli* [65]. Lipoprotein transporters, including the LolABCDE enzyme complex, have also been screened as novel targets. Recently, Nickerson et al. discovered a pyrrolpyrimidinedione inhibitor of LolCDE (G0507, Figure 1), which inhibits *E. coli* growth [66].

An additional target of interest is the Mla complex, which is responsible for maintaining the LPS-phospholipid asymmetry of the outer membrane. In a similar way to Lpt and Lol, this forms a multi-protein complex spanning the periplasm and is thought to remove mislocalised phospholipid from the outer leaflet, thus helping to maintain the integrity of the membrane barrier [67]. Mutations in the genes that encode the outer membrane MlaA protein, which closely associates with OmpC, a periplasmic protein MlaC, and an inner membrane ABC transporter MlaFEDB, have been shown to disrupt barrier function and could suggest these are potential targets for drug development. Interestingly, serial passage of a uropathogenic *E. coli* strain in the presence of arenicin-3 resulted in MlaC-associated resistance mutations, with the results supported by TraDIS data that showed enrichment of transposon mutations in mlaABCDEF in selected populations [68]. This suggests that MlaC could be the target for arenicin-3 and/or that enhanced activity of genes in this operon are a response to outer membrane disruption.

## 3. Peptidoglycan Layer and Inner Membrane

While the outer membrane represents a barrier to antibacterial compounds, it is only part of the mechanism of resistance in the cell wall complex. The peptidoglycan layer, which is found spanning the periplasmic space, is commonly used as a target for antibiotics due to its absence in humans. However, it has been hypothesised that the recycling of the peptidoglycan building blocks, often up-regulated in response to antibiotics, can activate secondary resistance mechanisms [69]. This includes the displacement of the UDP-MurNac-pentapeptide by anhydromuropeptides that are accumulated during β-lactam treatment [69]. In turn, this can disrupt the AmpR gene repression, causing the up-regulation of the β-lactamase AmpC and altered regulation of the MexEF efflux pump, which will be discussed in Section 5 [69].

There have been serious concerns over increasing reports of resistance to antibiotics that target the peptidoglycan layer. One approach to negate such resistance mechanisms is to move upstream of the peptidoglycan layer and look at the synthesis of its components. A promising antibiotic target is the diaminopimelate (DAP) pathway [70,71,72,73,74]. The DAP pathway is responsible for the production of *meso*-diaminopimelate (*meso*-DAP) and l-lysine, which are critical building blocks for cell wall and protein synthesis [75,76,77]. Inhibition of enzymes in the DAP pathway could result in a two-pronged approach of inactivation, effectively combining two modes of action, and thus, circumventing resistance. Other upstream targets include the well-studied MurF enzyme, responsible for the ligation of the d-Ala-d-Ala peptide on the cross-linking of the peptidoglycan layer [78]. We have recently published a review that describes the current status of targeting these, and other upstream pathways [79].

Unlike the outer membrane, the inner membrane is a phospholipid bilayer containing several enzymes responsible for energy metabolism and other important cellular processes [80]. Aside from any direct role in mediating resistance in GNB, especially working in synergy with the outer membrane, it remains a postulated target as it is the site of action for polymyxins [81].

## 4. Porins

The outer membrane provides a high level of protection against unwanted substances; however, it also limits the passive uptake of nutrients. To overcome this, bacteria use water filled pores, known as porins, to span the membrane, allowing the movement of size-specific hydrophilic compounds [82]. Porins consist of a ß-barrel structure, with hydrophobic amino acids facing into the outer membrane, and hydrophilic amino acids facing inwards, creating a pore to attract and allow transport of hydrophilic molecules across the membrane [83]. Porins can be monomeric, but are often found in trimers to enhance stability, with each unit hypothesised to function independently [83]. Porins are also known to form part of a tripartite structure of efflux pump proteins, which will be discussed in Section 5.

While there are porins that allow the transport of specific solutes, general diffusion channels are the ones most commonly associated with antibiotic resistance [82,84]. It is important to note that by some definitions, *P. aeruginosa* does not have a general diffusion porin, with the major OMP, OprF, being considered an outer membrane channel [85]. Nevertheless, OprF and other similar channels will be referred to as porins here.

Porin-mediated antibiotic resistance can be as simple as having a limited number of porins within the bacterial genome or controlling expression of selected porins. In a clinical isolate of *K. pneumoniae*, a nonsense mutation resulted in a translation error in the OmpK36 porin, compensated by up-regulation of other selective porins to maintain essential transport functions [86]. This is believed to have contributed to the development of resistance to all carbapenems tested [87]. The intrinsic resistance of *P. aeruginosa* is partly attributed to the more stringent exclusion criteria of its main porins such as OprF, which limits the penetration of antibiotics into the cell [88,89].

Structural modifications to porins can also confer antibiotic resistance. One example is the in-frame deletion of loop 7 of the *P. aeruginosa* OprD porin that results in carbapenem resistance, while still retaining its ability to transport arginine [90]. In *Salmonella enterica* serotype Typhimurium, changes in disulphide bond formation within the periplasm upon exposure to oxidative stress can regulate the opening and closing of the outer membrane porins OmpA and OmpC, resulting in increased resistance to β-lactams [91]. In *K. pneumoniae*, a premature stop codon in the OmpK35 porin is proposed to prevent the insertion into the membrane by blocking C-terminal anchoring [86]. This, combined with extended spectrum β-lactamases, resulted in carbapenem resistance in multiple Chilean clinical isolates [86]. This is also seen in other *Enterobacteriaceae* species [86]. While these porin alterations may decrease overall viability under non-selective conditions, the mutations will often be propagated under the selection pressure of otherwise lethal antibiotics.

As some of the major OMPs, porins have been implicated in other roles. For instance, OmpA (and its orthologue OprF in *P. aeruginosa*) is involved in the attachment of pathogenic bacteria to epithelial cells during infection [92]. It also plays a role in the stabilisation of the membrane, with its C-terminal non-covalently bound to the peptidoglycan layer [93,94]. Absence of OprF in *P. aeruginosa* can promote biofilm formation through the up-regulation of biofilm mediators such as bis-(3′-5′)-cyclic dimeric guanosine monophosphate [95]. While these roles do not contribute to resistance by blocking entry to the cell, these mechanisms can have indirect effects on the resistance profile of GNB by contributing to biofilm-dependent antibiotic tolerance.

**Figure 2:**
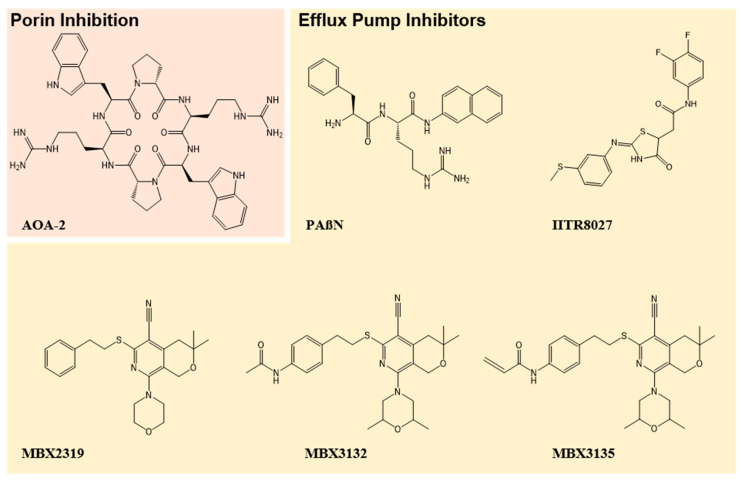
Porin and Efflux Pump Inhibitors. Porin (orange) and efflux pump (yellow) inhibitors. AOA-2 represents a promising inhibitor of the porin OmpA in *P. aeruginosa.* PAßN inhibits the MexAB-OprM efflux pump, whilst the efflux pump inhibitor IITR8027 has been used to re-sensitise *A. baumannii* to fluoroquinolones. MBX2319 acts upon the RND-type efflux pumps in *P. aeruginosa.* Its analogues, MBX3132 and MBX3135, were shown to have improved potency and stability.

These alternate functions of porins are of interest for drug development. Using computational techniques, Vila-Ferrēs et al. were able to design a cyclic hexapeptide that inhibits OmpA in both reference and clinical strains of *A. baumannii*, *E. coli* and *P. aeruginosa* [96]. While this inhibitor, AOA-2 (Figure 2), did not have antibacterial activity directly, it was able to reduce the attachment of bacteria to epithelial cells and subsequently reduce bacterial load in the lungs, spleen and blood culture in a murine sepsis model [96]. It is believed that this approach reduces the bacteria’s ability to evade the host’s immune system, allowing more time to clear the infection [96]. Nie et al. have written a timely review discussing non-antibiotic options for OmpA inhibition of MDR infections [97].

Rather than examining inhibition of porins themselves, a study by Haglan et al. examined the OMP assembly as a possible antibiotic target. The β-barrel structure of OMPs, including porins, are formed through an interaction network with β-barrel assembly machine (Bam) proteins [98]. This study looked at targeting the BamABCDE complex via the inhibition of BamD with a peptide sequence, derived from BamA. This resulted in growth defects in terms of colony size and colony forming units, as well as increased susceptibility to antibiotics that are typically unable to traverse the outer membrane [98]. As such, the interaction of BamD with BamA and porins such as OmpA, presents a promising new antibiotic target that would inhibit the OMPs from within [98]. There has also been considerable interest in targeting the outer membrane porins, such as TolC, associated with tripartite efflux pump systems as discussed below.

## 5. Efflux Pumps

The key to maintaining cellular homeostasis is to regulate the uptake of essential nutrients and solutes, whilst simultaneously expelling waste products and harmful substances. This process is governed by efflux pumps, which are membrane bound proteins that are found ubiquitously in bacteria [99,100]. Efflux pumps are responsible for the extrusion of a variety of solutes including fatty acids, heavy metals, antiseptics, detergents, virulence factors, toxins and most notably, antibiotics [101]. They can be specific to a single compound, class of antibiotics or have the ability to expel a number of structurally diverse classes of drugs (so called multi-drug efflux pumps), presenting a major problem in the fight against antibiotic resistance [101,102].

To extend through the GNB cell wall, some efflux pumps form tripartite assemblies from the inner membrane to the outer membrane [103]. This assembly consists of an inner membrane transporter protein, an OMP and a periplasmic membrane fusion protein [104]. Tripartite efflux pumps systems are associated mainly with the resistance-nodulation-cell division (RND) efflux superfamily [101], with some examples also from the ATP-binding cassette (ABC) family [105] and major facilitator superfamily (MFS) [103,104]. The most notable RND-type efflux pump is AcrAB-TolC from the *Enterobacteriaceae* family, covering important pathogens such as *E. coli, K. pneumoniae* and *Salmonella* spp. [106]. Homologous RND-type pumps are present in other GNB families, with relevant examples from *P. aeruginosa* (MexAB-OprM, MexCD-OprJ, MexEF-OprN and MexXY-OprM), *Campylobacter* spp. (CmeABC), *A. baumannii* (AdeABC) and *Neisseria* spp. (MtrCDE) [106]. Collectively, these tripartite RND efflux pumps have been shown to mediate resistance to a broad range of antibiotics, covering many classes, due to their overlapping substrate profiles [106,107,108,109]. Interestingly, the OMPs of tripartite assemblies are commonly the same or similar across different efflux pumps [103,110]. TolC is a well-characterised example and has been reported to contribute to resistance to many antibiotics and antiseptics including β-lactams, chloramphenicol, fluroquinolones, novobiocin, tetracycline and macrolides in the *Enterobacteriaceae* family [103,111,112]. The utilisation of TolC (or species equivalent) across various efflux pumps and bacterial families highlights the potential of OMPs as targets for the development of broad-spectrum compounds [103,104,113].

Providing clear evidence of the ability of efflux pumps to increase resistance to different drugs, OqxAB-TolC, a plasmid-borne efflux pump of the RND-family from *E. coli*, was transconjugated by Hansen et al. into *S. enterica* serotype Typhimurium, *K. pneumoniae, Enterobacter aerogenes* and *Kluyvera* sp., resulting in decreased susceptibility to ciprofloxin, olaquindox and chloramphenicol [114]. This was also shown for *E. cloacae*, in which genetic manipulation of the RND efflux pump AcrB linked its activity to both virulence and resistance to multiple antibiotic classes [115]. Reported mutations of efflux pump genes, such as those encoding the Mex efflux pumps from *P. aeruginosa*, have illustrated that antibiotic susceptibility is able to be restored, providing a possible avenue for the reinvigoration of current antibiotics [108].

Regulation of efflux pumps is complex and tightly controlled by a cross-talking set of regulatory pathways that involve the utilisation of TCSs and/or positive or negative transcriptional regulators to respond to a range of stimuli [116,117]. Common examples of TCSs that regulate the expression of MDR efflux pumps are AdeSR in *A. baumannii*, CpxAR in *Enterobacteriaceae* and AmgRS in *P. aeruginosa*. In the context of an antibiotic stress response, TCSs induce overexpression of efflux pump genes to ensure the removal of the target antibiotic [118]. The regulation of AcrAB-TolC in *E. coli, Salmonella* spp. and *Klebsiella* spp. is achieved by repressors and global transcriptional regulators of efflux pump associated genes [106]. In this instance, a member of the TetR family of transcriptional repressors, AcrR, is responsible for the prevention of overexpression of *acrAB* under normal growth conditions. Therefore, mutations in *acrR* result in the overexpression of AcrAB-TolC, leading to MDR phenotypes in *E. coli* and *Salmonella* isolates [119,120]. AcrR has also been identified as a repressor of other regulatory complexes, including MarAB and SoxS, as summarised by Ferrand and colleagues [121]. Mutations in another regulatory gene, *mexR*, have been shown to increase expression of the associated MexAB-OprM efflux system in *P. aeruginosa* isolates [122]. Other similar repressors, including RamR and OqxR, have also been linked to efflux pump regulation and overexpression, resulting in antimicrobial resistance for multiple species [121]. Environmental factors can also stimulate the overexpression of efflux pumps and can include oxidative stress, antibiotic application as well as the presence of specific ligands. The latter is evident in *E. coli* with the multiple antibiotic operon, Mar [106,123]. Furthermore, either direct mutations or mutations in the Tet-R family regulator of the MFS family efflux pump *smvA* in *K. pneumoniae*, *P. mirabilis, E. cloacae* and *S. enterica* serotype Typhimurium are associated with increased biocide resistance, including to chlorhexidine [124,125,126] and acriflavine [127]. Efflux pump regulatory pathways have been noted to be interwoven into many other regulatory processes that promote bacterial virulence such as biofilm formation, quorum sensing and membrane permeability [110,128,129].

Due to their broad substrate specificity, efflux pumps have long been considered a promising target for adjuvant development, aiming to re-sensitise bacteria to a number of antibiotics. The relatively recent structural elucidation of major tripartite pumps in MDR clinical pathogens has assisted in the development and optimisation of inhibitors. The most well-characterised efflux pump inhibitor (EPI) is phenylalanine-arginine beta-naphthylamide (PAßN) (Figure 2), which inhibits the MexAB-OprM efflux pump and related RND-family pumps in clinically relevant pathogens, including *P. aeruginosa* [130,131]. However, PAßN has limited clinical use as the high concentrations required for treatment results in off-target cytotoxicity to the host [132]. EPIs that function through dissipation of the proton motive force, including cyanide *m*-chlorophenylhydrazine (CCCP), are also cytotoxic [133]. Whilst these compounds may be used as research tools for in vitro antibiotic testing, neither are useful for clinical use. Unfortunately, this drawback seems to apply to most EPI development, with no inhibitors successfully completing clinical trials to date. Recently, a promising small molecule inhibitor (IITR8027, Figure 2) of proton driven *A. baumannii* efflux pumps has re-sensitised MDR bacteria to ciprofloxacin, despite displaying no antibacterial activity on its own [134]. Additionally, IITRR8027 showed no cytotoxicity at the reported minimum effective concentration for ciprofloxacin potentiation [134]. The novel pyranopyradine inhibitor MBX2319 (Figure 2) has been shown to potentiate current antibiotics through inhibition of the AcrAB efflux pump in *E. coli*; however, the lack of potency and stability were major drawbacks [135]. A subsequent study used MBX2319 as a scaffold to produce analogues with enhanced potency and stability [136]. Analogues MBX3132 and MBX3135 (Figure 2) were able to inhibit more than one type of RND efflux pump in *E. coli*, whilst retaining activity against a panel of *Enterobacteriaceae* [137]. Such broad-spectrum efflux pumps inhibitors without associated toxicity may have significant value in potentiating the efficacy of multiple classes of antibiotics.

## 6. Combinatorial Approaches

Resistance phenotypes in bacteria can be due to the presence of single genes (e.g., carbapenemases) or, more commonly, a combination of different resistance mechanisms. For instance, in the case of carbapenem resistance, porin down-regulation results in resistance to imipenem but only in combination with increased efflux will it result in resistance to both meropenem and doripenem [138]. Often loss of a specific porin is also combined with up-regulation of an enzyme, such as AmpC, which is capable of degrading the carbapenem, albeit with relatively poor turnover [139]. In *P. aeruginosa*, mutations in either the *mexT* or *mexS* genes can decrease porin OprD expression, whilst concurrently increasing MexEF-OprN expression, which leads to resistance to imipenem, quinolones and chloramphenicol [140]. Many of these intrinsic mechanisms are coupled with other adaptive resistance mechanisms, for example carbapenemase expression, AmpC overexpression and target alteration [141]. For instance, a MDR *E. coli* strain has been found to have decreased permeability to and increased efflux of ciprofloxacin, coupled with a mutation in DNA gyrase, further increasing its resistance [103]. Transient or constitutive up-regulation of efflux pump expression may be a common first response in many bacteria to treatment with an antibiotic and be an essential stepping stone to the development of target-site resistance mutations [142]. Biofilms also play a key role in phenotypic antibiotic resistance, which has been extensively covered and will not be discussed here [143,144,145,146,147,148]. It is this interconnected network of resistance mechanisms that allows for bacteria to become resistant to an extensive range of antibiotics, and as such, development of effective therapeutics should aim to take a multi-targeted approach.

A combinatorial approach of inhibitors may be the key to overcoming antibacterial resistance. Making antibiotic analogues that are efflux resistant to prevent the initial efflux-mediated resistance response may be an attractive approach to reducing the emergence of resistance. Similarly, the combination of a membrane permeabilising agent with an efflux pump inhibitor may have benefits in lowering EPI concentration to minimise cytotoxicity and potentiate a broader range of antibiotic agents that would otherwise have been sensitive to efflux. Utilising the EPI PAßN and the membrane permeabiliser PMBN, Ferrer-Espada et al. were able to improve the activity of azithromycin by 2000-fold with 1 μg/mL PMBN [149]. Interestingly, this could not be achieved with the EPI alone [149]. Ideally, if the right efflux pump inhibitor was developed, mammalian toxicity could be significantly reduced by lowering the dose required with compounds like PMBN.

**Figure 3:**
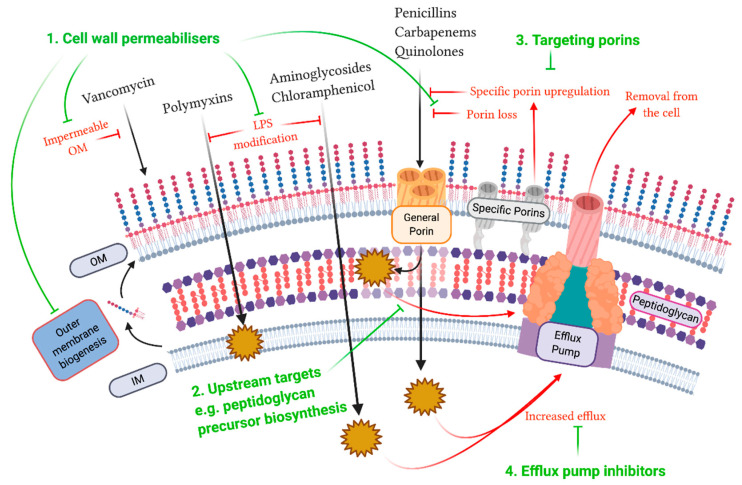
Novel Approaches to Bypass Resistance Mechanisms. Shown in green are the potential approaches for overcoming antibiotic resistance mechanisms associated with the cell wall in Gram-negative bacteria that have been discussed in this review. (1) The first of approach is using compounds to disrupt and permeabilise the cell wall, including targeting outer membrane biogenesis and transport or by using non-antibacterial polymyxin compounds that increase susceptibility to current antibiotics. (2) Targets upstream of the peptidoglycan layer, such as the biosynthesis of peptidoglycan precursors, represent potential new modes of action that may be able to circumvent pre-existing resistance mechanisms. (3) Porin inhibition is an attractive antibiotic target, which can reduce bacterial virulence, thereby aiding in clearance of the infection by the immune system. (4) As almost all antibiotic classes are susceptible to efflux, efflux pump inhibitors aim to block the extrusion of antibiotics, thus increasing their active concentration within the cell. Figure was generated using BioRender.com.

Further combinatorial approaches have focused on the utilisation of adjuvant compounds. Such adjuvants typically have no antibacterial activity on their own but can result in synergistic responses in combination with current antibiotics [150]. Several studies have shown the potential for adjuvants to potentiate antibiotics that are specific to Gram-positive bacteria to treat resistant strains of GNB [151,152]. This was achieved in one paper by adding anthracyclines to rifampicin and linezolid, whereby the combination resulted in increased susceptibility to both *A. baumannii* and *E. coli* [151]. Any of the aforementioned approaches that target the cell wall have the potential to be used in combination to revitalise our current antibiotic arsenal or to act as a stand-alone antibacterial agent (Figure 3).

## 7. Conclusions

AMR represents a major threat to our ability to treat and prevent common infections. A major contributor to this resistance is the intricate membrane system of GNB, which is a potent barrier to the uptake of existing and new antibacterial agents. This includes impermeability due to the outer membrane composition and porin selectivity, as well as increased efflux from the cell. Furthermore, by understanding membrane synthesis and maintenance, we can examine potential new avenues for the development of adjuvant therapies. Here, we have described several examples of these, including membrane permeabilisation agents, targeting porin alternate functions and efflux pump inhibitors, which can be employed as new modes of action alone or in combinatorial treatments. By highlighting this as a challenge-led approach, as we have done here, we can open up avenues to revitalise our current antibiotic arsenal as well as stimulate the development of new agents to tackle the global antibiotic resistance crisis.
